# Efficacy of Pregabalin and Duloxetine in Patients with Painful Diabetic Peripheral Neuropathy (PDPN): A Multi-Centre Phase IV Clinical Trial—BLOSSOM

**DOI:** 10.3390/ph16071017

**Published:** 2023-07-18

**Authors:** Martin Rakusa, Iris Marolt, Zorica Stevic, Sandra Vuckovic Rebrina, Tatjana Milenkovic, Adam Stepien

**Affiliations:** 1 Division of Neurology, University Medical Centre Maribor, 2000 Maribor, Slovenia; 2 Faculty of Medicine, University of Maribor, 2000 Maribor, Slovenia; 3 Outpatient Diabetes Clinic, Community Health Centre Koper, 6000 Koper, Slovenia; 4 Neuropathy Center, Neurology Clinic, Clinical Center of Serbia, 11000 Belgrade, Serbia; 5 School of Medicine, University of Belgrade, 11000 Belgrade, Serbia; 6 University Clinic Vuk Vrhovac, Clinical Hospital Merkur, 10000 Zagreb, Croatia; 7 University Clinic of Endocrinology, Diabetes and Metabolic Disorders, 1000 Skopje, North Macedonia; 8 Faculty of Medicine, Ss. Cyril and Methodius University in Skopje, 1000 Skopje, North Macedonia; 9 Department of Neurology, Military Institute of Medicine—National Institute of Science, 04-052 Warsaw, Poland

**Keywords:** neuropathic pain, diabetic polyneuropathy, duloxetine, pregabalin, randomised clinical trial

## Abstract

Introduction: Our trial (ClinicalTrials.gov Identifier: NCT04246619) evaluates the efficacy of two generic medications, pregabalin and duloxetine, for treating pain in PDPN patients. Methods: The patients were randomised either into the pregabalin (99) or the duloxetine (102) arm. Pain was evaluated using the DN-4 questionnaire, and visual analogue scales (VASs, 0–100 mm) were used to measure the average pain intensity (API), worst pain intensity (WPI) in the last 24 h and current pain intensity (CPI). Results: The proportion of patients with a clinically significant improvement in the API at Week 12 was 88.3% [CI 81.7%, 94.8%] in the pregabalin arm and 86.9% [CI 76.7%, 97.1%] in the duloxetine arm. After 12 weeks, the CPI, API, and WPI decreased by −35.3 [−40.5, −30.0], −37.0 [−41.4, −32.6], and −41.6 [−46.6, −36.5] in the pregabalin arm, and by −35.0 [−39.2, −30.7], −36.9 [−41.5, −32.3], and −40.0 [−44.8, −35.2] in the duloxetine arm (all in mm, all *p* < 0.001). Conclusion: Our results demonstrate that pregabalin and duloxetine are effective medications for treating pain in PDPN in more than 86% of all randomised patients.

## 1. Introduction

Diabetic polyneuropathy (DPN) is the most common cause of neuropathy worldwide, affecting approximately half of the patients with diabetes mellitus (DM) [1]. Painful diabetic polyneuropathy (PDPN) is a long-term complication of DM, characterised by peripheral nerve damage and neuropathic pain. It is a common cause of morbidity in diabetic patients [2].

The prevalence of PDPN varies across studies, affecting between 13% and 63% of patients with DM, depending on the study population and diagnostic criteria [3,4,5]. The risk factors associated with PDPN differ between studies and include increased age, duration of DM, and poor glycaemic control [3,4,5,6]. In addition, some studies suggest that other comorbidities, such as hypertension and dyslipidaemia, may play an important role in developing PDPN [7,8].

PDPN can be debilitating, impacting patients’ physical, psychological, and emotional well-being [9]. PDPN is also associated with a significant decrease in patients’ quality of life and increased healthcare costs [5,6].

The effective management of PDPN requires a stepwise approach that includes accurate diagnostics, glycaemic control, education and counselling on foot care and safety measures, and symptomatic treatment of pain with medicines [4,10].

The efficacy of pregabalin and duloxetine has been extensively studied. Parsons and Li [11] included 11 placebo-controlled studies evaluating the effects of a flexible or fixed dose of pregabalin on PDPN. The authors reported that pregabalin effectively reduced moderate or severe pain associated with PDPN. Similarly, a study from the Cochrane Database of Systematic Reviews evaluated the efficacy of duloxetine in the treatment of PDPN. Lunn et al. [12] analysed eight randomised controlled trials with approximately 2800 patients. The results show that duloxetine effectively reduced pain in patients with PDPN compared to the placebo. Another meta-analysis demonstrated that pain severity can be reduced by 30% to 50% using pregabalin or duloxetine [13].

Due to the high prevalence of PDPN, there is a need for more affordable treatment options. Several reasons exist for conducting a multi-centre study with generic pregabalin and duloxetine to treat PDPN. First, such research can help determine their effectiveness in treating PDPN and identify potential side effects associated with their use. Secondly, it can help to identify which patients may benefit the most from this treatment and whether any factors may influence the effectiveness of the treatment such as age, gender, or severity of the condition. Additionally, it may provide valuable experience to physicians who are managing patients with PDPN and increase confidence in their use among patients and clinical bodies.

We conducted a multi-centre open-label study to evaluate the efficacy and safety of two generic drugs—pregabalin (Pregabalin Krka) and duloxetine (Dulsevia^®^). The objectives of the study are to assess the efficacy of both medicines in patients with PDPN; investigate their effect on pain and quality of life (QoL), depression symptoms, cognitive functions, sleep quality and daytime sleepiness; and assess their safety in patients with PDPN. Here, we present the efficacy and safety results of both drugs.

## 2. Results

### 2.1. Population

**Figure 1:**
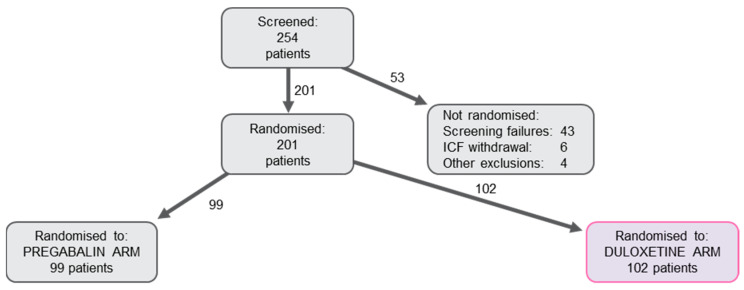
Patient disposition (FAS details).

Of the 254 patients who were screened, 201 (79.1%) were assigned to the study treatment; 201 patients were analysed for main safety endpoints. A total of 201 patients provided sufficient efficacy data for inclusion in the Full Analyses Set (FAS). Out of the 53 (20.9%) patients who were not randomised, 6 (2.4%) patients withdrew their Informed Consent Forms (ICFs), 43 (16.9%) patients were screening failures (SFs), and 4 (1.6%) were classified as other exclusions (3 patients due to the epidemic in Serbia; 1 patient was uncooperative). Out of the 201 randomised patients constituting the FAS, 158 (78.6%) completed the study (i.e., completed Visit 5), while 43 (21.4%) were prematurely excluded (Figure 1).

The majority of patients included in the study were female. Table 1 presents the demographic data of the patients included in the study. The majority of patients in both arms had comorbidities, with 90.9% in the pregabalin arm and 93.1% in the duloxetine arm. The most common comorbidities were vascular disorders and metabolism and nutrition disorders, with similar percentages in both arms. The other comorbidities listed in Table 1 were also present but with lower frequencies.

### 2.2. Efficacy on Acute Pain

The primary objective of our study was fulfilled by 88% of patients who had a clinically meaningful improvement in the 24 h-API after a 12-week treatment in the pregabalin arm (Table 2). In addition, 15% of patients in the pregabalin arm reported a clinically meaningful improvement (≥30%) in the CPI, 24 h-API, and 24 h-WPI in the first week of treatment (Table 2). In the following weeks, their number increased to 83% at the end of the clinical trial (Table 2). Similarly, the proportion of patients who reached a reduction from the baseline in the CPI, 24 h-API, and 24 h-WPI by ≥50% increased from the trial onset to 58% by Week 12 (Table 2). Around one-third of patients reported less than 10 mm of CPI, 24 h-API, and 24 h-WPI at the end of the clinical trial (Table 2). On average, the patients reported an absolute reduction of 35 mm in the CPI, 37 mm in the 24 h-API, and 42 mm in the 24 h-WPI at the end of the clinical trial (Table 3). Similar results were observed for the DN-4, with a relative reduction of 48% (Table 2).

Similarly, 87% of patients in the duloxetine arm had a clinically meaningful improvement in the 24 h-API after 12 weeks (Table 2). A clinically meaningful improvement in the CPI, 24 h-API, and 24 h-WPI was already observed after the first week of treatment for 24% of patients (Table 2). This number increased during the trial and reached 82% in Week 12 (Table 2). In addition, the proportion of patients who reached a reduction from the baseline in the CPI, 24 h-API, and 24 h-WPI by ≥50% increased from the trial onset, and by Week 12, 68% of patients had significantly less pain (Table 2). At the end of the clinical trial, more than 20% of patients reported less than 10 mm of CPI, 24 h-API, and 24 h-WPI (Table 2). On average, the patients reported an absolute reduction of 35 mm in the CPI, 37 mm in the 24 h-API and 40 mm in the 24 h-WPI at the end of the clinical trial (Table 3). Similar results were observed for the DN-4, with a relative reduction of 48% (Table 2).

### 2.3. Efficacy on Subacute Pain

Around 80% of patients in the pregabalin arm had a reduction in the 4 wk-API and 4 wk-WPI by at least 30% and/or did not exceed 30 mm between Week 8 and Week 12 (Table 2). A total of 56% of patients reached a reduction from the baseline in the 4 wk-API and 4 wk-WPI by 50% or more at Week 12 (Table 2), and approximately 18% of patients were without pain (<10 mm on VAS) (Table 2). The mean relative changes in the 4 wk-API and 4 wk-WPI increased during the trial, and by Week 12, reached 54% and 52%, respectively (Table 3), with a mean absolute change of 39 in the 4 wk-WPI and 32 in the 4 wk-API at Week 12.

Our results also indicate reduced subacute pain in the duloxetine arm. Almost 80% of patients had a reduction in the 4 wk-API and 4 wk-WPI by at least 30% and/or did not exceed 30 mm between Week 8 and Week 12 (Table 2). More than two-thirds of the patients reached a reduction from the baseline in the 4 wk-API and 4 wk-WPI by 50% or more at Week 12 (Table 2), and approximately 15% of patients were without pain (<10 mm on VAS) (Table 2). The mean relative change in the 4 wk-API was 60% and 56% in the 4 wk-WPI at the end of the clinical trial (Table 3), with a mean absolute change of 41 in the 4 wk-WPI and 36 in the 4 wk-API at Week 12. Although the proportion of patients who benefitted from treatment in the duloxetine arm increased until the end of the clinical trial, the proportions were similar by Week 8 and Week 12.

### 2.4. Adverse Events

In both groups, approximately 32% of patients experienced non-serious AEs. In the pregabalin arm, the most commonly reported AEs were nervous system disorders and ear and labyrinth disorders. In the duloxetine arm, the most commonly reported AEs were gastrointestinal disorders and nervous system disorders (Table 4). There were five non-related and one related serious AE. For a detailed list of AEs per treatment, see Supplementary Tables S2 and S3.

## 3. Discussion

We report the results from an open-label multi-centre clinical trial in which we assessed the efficacy and safety of Pregabalin Krka and Dulsevia^®^. The patients in both arms reached primary and secondary goals. In addition, almost 90% of the patients treated with either drug had a clinically significant reduction in acute pain (Table 2 and Table 3).

In order to exclude other known causes of painful polyneuropathy (e.g., abnormal thyroid-stimulating hormone (TSH) concentrations, excessive alcohol consumption, vitamin B12 and folic acid deficiency, uncontrolled type 2 diabetes mellitus), we performed an extensive laboratory evaluation at the beginning of the clinical trial.

### 3.1. Efficacy on Acute Pain

Our study results are similar to those of previously published research, which demonstrated the beneficial effect of pregabalin on PDPN. Derry et al. recently performed a meta-analysis of the double-blind clinical trials evaluating the efficacy of pregabalin [14]. Their results show that around 60% of patients treated with 600 mg of pregabalin had at least a 30% pain intensity reduction, and around 40% had at least a 50% pain intensity reduction. Similar results were obtained in open-label studies. The total pain reduction measured using a VAS was between 25% and 51% [15].

In our study, patients were recruited from several different countries, similar to the placebo-controlled trials. In addition, compared to the placebo-controlled trials, we evaluated pain parameters such as the API, CPI, and WPI [14].

In addition, our results demonstrate that patients in the duloxetine arm had significantly less CPI, 24 h-API, and 24 h-WPI during and at the end of the clinical trial than at the beginning (Table 2 and Table 3). When we compare our results with the results from the meta-analysis, the proportion of patients who benefitted is higher in our study (87% vs. 70%) [12]. We also found more patients with pain reduced by at least 50% compared to previous trials (Table 2). Lunn et al. performed a meta-analysis including eight clinical trials [12]. The proportion of patients with a pain reduction of ≥50% was around 50% for patients treated with a fixed dose of 60 mg or 120 mg of duloxetine. However, we found a similar proportion of reductions in CPI, 24 h-API, and 24 h-WPI (Table 3) at the end of the clinical trial [12]. There are two main differences between our study and previous studies. First, in the previous studies, duloxetine was compared to the placebo; we therefore cannot exclude the possibility that slightly higher results in our study may partly be due to the placebo effect. On the other hand, in the open-label trials where they compared the effects of duloxetine and pregabalin, pain was reduced by 30% and 40%, as measured using a VAS [15,16], which is similar to our results.

### 3.2. Efficacy on Subacute Pain

In contrast to the previous studies, we also evaluated the effect of pregabalin on subacute pain. Patients from the duloxetine arm in our study experienced a significant improvement in the 4 wk-API and 4 wk-WPI (Table 2 and Table 3). Such results can be expected since we have already demonstrated an acute pain reduction. In addition, the effect of pregabalin is dose dependent [17], and patients in our study increased their dosage until the end of the clinical trial (Table 5).

However, directly comparing our results with previous studies is impossible. In previous studies, patients evaluated their pain in one week [18] or as part of the SF-36. However, the SF-36 bodily pain domain provides limited information on pain intensity in a four-week period and does not differ between neuropathic and nociceptive pain [19].

In our study, patients from the duloxetine arm also reported improved chronic pain (Table 2 and Table 3). However, most of the previous studies evaluated 24 h or immediate pain [16]. Therefore, we cannot compare our results directly. Some studies also included the SF-36, in which the bodily pain domain unspecifically evaluates pain for the last four weeks [12,20,21,22]. The results demonstrate that the patients who received duloxetine had higher scores 12 weeks after treatment and had reduced pain; this is in line with our results. In addition, our results contribute to a better understanding of the efficacy of duloxetine. However, the patients in our study had similar subacute pain 8 and 12 weeks after the treatment. A possible reason could be a similar dose of duloxetine (Table 5).

There are a few studies in which authors evaluated the efficacy of duloxetine and pregabalin in patients with PDPN with regard to disease characteristics; e.g., Ziegler et al. analysed data from three randomised control trials and found no associations between the efficacy of duloxetine and the patient’s age, type or duration of diabetes, severity of diabetic neuropathy, HbA1c baseline levels, or baseline insulin levels. The only significant interaction was related to pain severity, where patients with more severe pain had better treatment outcomes [23]. Similarly, Alexander et al. proposed that the efficacy of pregabalin can be predicted based on the response to treatment at Week 4 rather than based on the baseline characteristics [24]. Our study did not try to predict efficacy.

### 3.3. Safety and Tolerability

The patients included in this study tolerated pregabalin and duloxetine well. Approximately two-thirds of the patients did not report any AEs, and only 3% had serious AEs.

Similar to previous studies, the most common treatment-related AEs in the pregabalin arm were somnolence and vertigo (Supplementary Table S2) [14,18]. Five percent of patients had pregabalin emergent AEs (Table 4), which is lower than what was reported in the meta-analysis of the double-blind clinical trials [14].

One patient who received pregabalin completed suicide. There were five deaths in the previous trials [25]. It is known that pregabalin may induce suicidal ideation and changes the mood in patients with neuropathic pain and a history of depression [26,27]. Therefore, we need to warn patients before treatment onset.

In the duloxetine arm, the most common treatment-related AEs were nausea, dizziness, and somnolence (Supplementary Table S3), similar to the AEs reported in the meta-analysis [28].

Due to therapy-emergent AEs, 13% of patients discontinued the clinical trial, similar to the previous studies [29]. The most common treatment-emergent AE leading to study discontinuation in the pregabalin arm was peripheral oedema (2.0%); all other related AEs were recorded for a single patient. In the duloxetine arm, the most common treatment-emergent AEs leading to study discontinuation were nausea (2.9%), somnolence (2.9%), and decreased appetite (2.9%). In addition, we found that both drugs had good compliance. Around 90% of patients had compliance exceeding 80% at Week 12 despite the high treatment dosages (Table 5).

While our clinical trial was not explicitly structured to assess the comparative efficacy of the two drugs, it is essential to take into account the potential side effects when determining the optimal treatment for individual patients. For instance, patients with PDPN who also have a history of mood disorders might experience adequate pain relief from either medication. However, prior research indicated that while duloxetine could potentially ameliorate depressive symptoms [23], pregabalin might exacerbate mood disorders [26,27]. Conversely, for a patient who is susceptible to nausea, pregabalin might prove to be a more suitable choice than duloxetine (Supplementary Tables S2 and S3). Therefore, the decision on the therapeutic choice should be tailored based on a comprehensive understanding of the patient’s unique clinical situation and medical history.

### 3.4. Strengths and Limitations

Our study has several strengths. First, it is a multi-centre clinical trial. Second, we included a homogeneous sample of patients with type 2 DM and used a dynamic treatment regime. In this way, we replicated a real-life situation. In addition, we assessed changes in subacute neuropathic pain, which has not been carried out before.

As with every study, ours had some limitations as well. First, it was not a double-blind clinical trial, and the achievement of better results compared with the placebo-controlled trials may be partly due to the placebo effect. Another reason may be a different population. However, with well-known benefits from the previous trials, it would be unethical to conduct a placebo-controlled study. Another drawback is that all the patients in our study were white. Therefore, we may not be able to expand our results to other races, and the study results are within our sample’s limits.

## 4. Materials and Methods

This study is an interventional, open-label, prospective, international, multi-centre, phase IIIb (Poland)/IV (Croatia, North Macedonia, Serbia, and Slovenia) clinical study. Both generic medications are manufactured by Krka, d. d., Novo mesto, Slovenia. The complete protocol is available at clintrials.gov (ClinicalTrials.gov Identifier: NCT04246619) [30]; for detailed explanation of inclusion and exclusion criteria, see Supplementary Table S1.

### 4.1. Population

The protocol originally aimed to have 280 patients complete the efficacy assessments, with a per-protocol population of 160 patients. We assumed a 40% dropout rate for the sample size of 280 participants. The minimum required number of treated participants per protocol was 160. However, due to recruitment challenges during the COVID-19 pandemic, fewer patients were enrolled, and when the required number of patients was reached, we terminated the clinical trial with no effect on sample power. Nonetheless, the study could still provide meaningful results with the same statistical power as initially intended, justifying the early termination.

### 4.2. Protocol

We included men and women aged 18–85 years with PDPN caused by type 2 DM. Acute pain had to be scored ≥4 on the Douleur Neuropathique questionnaire (DN-4) [31], and the average pain intensity in the last 24 h (24 h-API) had to be ≥40 mm on the 0–100 mm Visual Analog Scale (VAS), with 0 mm corresponding to ’no pain’, and 100 mm corresponding to ‘worst possible pain’. All patients signed informed consent forms, and regulatory agencies in each participating country approved the study.

The clinical trial lasted 12 weeks, similar to previous clinical trials aimed at assessing the efficacy and safety of pregabalin [14] and duloxetine [12]. Acute pain was defined as pain at Visit 1, and subacute pain was defined as pain from Week 4 to Week 12.

During the screening visit (Visit 1), the investigator conducted laboratory analyses (concentrations of the thyroid-stimulating hormone (TSH), vitamin B12, folic acid, glucose, glycated haemoglobin (HbA1c), pregnancy test for women of childbearing potential), a PDPN assessment (DN-4), a cognitive assessment using Montreal Cognitive Assessment (MoCA) [32], a medical history evaluation (medical/surgical history and concomitant diseases, previous or existing therapy of pain in PDPN, concomitant medications, habits), and a pain evaluation.

On Visit 2 (baseline visit; 0–7 days after Visit 1), the investigator assessed the results of the laboratory tests, vital signs, pain intensity, previous analgesic therapy, and concomitant medications.

Patients who met all inclusion criteria and did not meet any exclusion criteria were randomly assigned to the pregabalin arm (Pregabalin Krka in Slovenia, Pragiola^®^ in all other countries) or the duloxetine arm (Dulsevia^®^ in all countries). The investigator additionally assessed the QoL (36-Item Short Form Survey Instrument, SF-36) [19], sleep quality and daytime sleepiness (the Insomnia Severity Index, ISI) [33] and Epworth Sleepiness Scale (ESS) [34], depression (Major Depression Inventory, MDI) [35], and adverse events.

At Week 1 (7 ± 3 days after Visit 2), the investigator contacted the patients by phone (Phone Call 1) to evaluate pain intensity and adverse events recorded in Patient Diary 1.

At Visit 3 (14 ± 3 days after Visit 2), compliance monitoring was performed, pain intensity was evaluated, and concomitant therapy, vital signs, and adverse events were assessed. The doses of the investigational medicinal product (IMP) were adjusted.

At Week 6 (28 ± 3 days after Visit 3), the investigator contacted patients by phone (Phone Call 2) to evaluate pain intensity and adverse events, which were recorded in Patient Diary 2.

At Visit 4 (42 ± 3 days after Visit 3), the investigator conducted a pregnancy test for women of childbearing potential, conducted compliance monitoring, assessed concomitant medications, measured vital signs, evaluated pain intensity, adjusted the doses of IMP, and assessed adverse events. The investigator collected Patient Diary 2.

At Visit 5 (28 ± 3 days after Visit 4), the investigator assessed QoL, sleep quality and daytime sleepiness, depression, cognition, and PDPN using various questionnaires. Pain intensity was evaluated using VAS and DN-4, and adverse events were assessed. A pregnancy test for women of childbearing potential was also conducted. Completing all procedures at Visit 5 marked the end of the patient’s involvement in the clinical study.

The investigators evaluated current pain intensity (CPI), average pain intensity in the last 24 h (24 h-API), and worst pain intensity in the last 24 h (24 h-WPI) at all visits and phone calls according to VAS. They also evaluated average pain intensity (4 wk-API) and worst pain intensity (4 wk-WPI) in the last 4 weeks at Visit 4 and Visit 5.

### 4.3. Treatment Protocol

The full treatment protocol is presented in Supplementary Figure S1.

### 4.4. Efficacy and Safety Evaluation

Efficacy was evaluated using several parameters, such as average pain intensity (according to VAS) in the last 24 h and 4 weeks, current pain intensity, worst pain intensity in the last 24 h and 4 weeks, assessment of peripheral diabetic neuropathy (using DN-4 questionnaire), assessment of cognition (using MoCA questionnaire), assessment of the QoL (using SF-36 questionnaire), assessment of sleep quality and daytime sleepiness (using ISI and ESS questionnaires), and assessment of depression (using MDI questionnaire).

The primary study endpoint was to determine the proportion of patients with PDPN who showed a clinically meaningful improvement in pain. Improvement in pain was considered as clinically meaningful if the reduction in 24 h-API was equal to or more than 30% in comparison to the baseline and/or if the 24 h-API did not exceed 30 mm after the 12-week treatment.

The secondary efficacy endpoints (in relation to pain evaluation) were used to determine the proportion of patients with a clinically meaningful improvement in CPI, 24 h-API, and 24 h-WPI separately during all time points; 4 wk-API and 4 wk-WPI at Weeks 8 and 12; mean absolute change from baseline for all time points in CPI, 24 h-API, and 24 h-WPI separately, and in 4 wk-API and 4 wk-WPI for Weeks 8 and 12; the proportion of patients with reduction in pain by 50% or more in CPI, 24 h-API, and 24 h-WPI on all time points and in 4 wk-API and 4 wk-WPI at Weeks 8 and 12; the proportion of patients with pain not exceeding 10 mm (via VAS) in CPI, 24 h-API, and 24 h-WPI at Weeks 6, 8, and 12, and in 4 wk-API and 4 wk-WPI at Weeks 8 and 12; and to determine the mean difference in the DN-4 questionnaire from baseline and Week 12.

Safety was evaluated by recording and assessing adverse events (AEs) throughout the study, up to Visit 5. In addition, vital signs (heart rate, office blood pressure) were measured at each study visit, while clinical laboratory tests were conducted at Visit 1, evaluating the concentrations of TSH, vitamin B12, folic acid, glucose, and HbA1c. Laboratory analyses served to verify eligibility of patients and exclude other possible causes of painful polyneuropathies. No further analyses between laboratory parameters and pain were performed.

### 4.5. Statistical Analysis

The Full Analysis Set (FAS) was used for primary and secondary efficacy evaluations and safety analyses. However, due to missing values in the patient records, any required data not available for a given patient at any time point from Visit 2 onwards were considered missing data regardless of the reason for the missing data (e.g., measurement not performed, premature exclusion, or input error). To address this, multiple imputation (MI) was employed, using specific statistical inference methods designed for MI estimates.

Summary statistics were generated for categorical and numerical variables, including the number of patients/observations, frequencies, percentages, mean, median, standard deviation, minimum and maximum, and first and third quartiles. Microsoft Visual Basic for Applications and IBM SPSS 28.0 were utilised for these calculations.

## 5. Conclusions

The results of our study offer valuable insights into the effectiveness and safety of two generic drugs, Pregabalin Krka and Dulsevia^®^, in managing PDPN. Despite both drugs demonstrating efficacy in managing PDPN, the frequency and nature of adverse reactions might differ between them. This difference could play a role in determining the optimal therapy for each individual patient. This information can potentially benefit doctors and patients by setting expectations for treatment outcomes.

## Figures and Tables

**Table 1:** Patient physical characteristics, habits, and comorbidities.

		Pregabalin Arm	Duloxetine Arm
	n	99	102
Sex	Female	56 (56.6%)	61 (59.8%)
Age (years)	Mean ± SD	64.4 ± 9.1	63.4 ± 10.1
Height (cm)	Mean ± SD	168.3 ± 9.7	168.6 ± 9.6
Weight (kg)	Mean ± SD	85.7 ± 16.2	83 ± 13.4
BMI (kg/m^2^)	Mean ± SD	30.3 ± 5.6	29.3 ± 4.8
Time from first diagnosis of type 2 DM (years)	Mean ± SD	12.7 ± 8.8	12.7 ± 9.1
Smoking	No	72 (73%)	71 (70%)
Yes		27 (27%)	31 (30%)
	Ex-smoker	11 (11.1%)	8 (7.8%)
	Occasional smoker	2 (2%)	4 (3.9%)
	Regular smoker	14 (14.1%)	19 (18.6%)
Alcohol consumption	No	82 (83%)	89 (87%)
Yes		17 (17%)	13 (13%)
	Occasionally drinks	16 (16.2%)	11 (10.8%)
	Regularly drinks	1 (1%)	2 (2%)
	Other	0 (0%)	0 (0%)
Drug abuse	No	99 (100%)	102 (100%)
Patients with comorbidities		90 (90.9%)	95 (93.1%)
Vascular disorders		72 (72.7%)	78 (76.5%)
Metabolism and nutrition disorders		56 (56.6%)	53 (52%)
Cardiac disorders		12 (12.1%)	20 (19.6%)
Endocrine disorders		12 (12.1%)	15 (14.7%)
Gastrointestinal disorders		13 (13.1%)	13 (12.7%)
Musculoskeletal and connective tissue disorders		12 (12.1%)	12 (11.8%)
Eye disorders		7 (7.1%)	11 (10.8%)
Reproductive system and breast disorders		4 (4%)	9 (8.8%)
Nervous system disorders		6 (6.1%)	5 (4.9%)
Respiratory, thoracic, and mediastinal disorders		6 (6.1%)	4 (3.9%)
Renal and urinary disorders		5 (5.1%)	3 (2.9%)
Surgical and medical procedures		7 (7.1%)	0 (0%)
Blood and lymphatic system disorders		4 (4%)	1 (1%)
Hepatobiliary disorders		4 (4%)	1 (1%)
Psychiatric disorders		0 (0%)	3 (2.9%)
Immune system disorders		1 (1%)	1 (1%)
Infections and infestations		2 (2%)	0 (0%)
Investigations		1 (1%)	1 (1%)
Skin and subcutaneous tissue disorders		1 (1%)	1 (1%)
Ear and labyrinth disorders		1 (1%)	0 (0%)
Injury, poisoning, and procedural complications		0 (0%)	1 (1%)
Neoplasms		1 (1%)	0 (0%)

**Table 2:** Efficacy in pregabalin and duloxetine arms.

	Pregabalin Arm	Duloxetine Arm
	(*n* = 99)	(*n* = 102)
Patients with clinically meaningful improvement in 24 h-API ^1^	88.3%; [81.7%, 94.8%]	86.9%; [76.7%, 97.1%]
Patients with clinically meaningful improvement (≥30%) in CPI ^2^, 24 h-API ^1^, and 24 h-WPI ^3^		
Week 1	14.7%; [7.5%, 22.0%]	23.5%; [15.2%, 31.9%]
Week 2	31.9%; [22.5%, 41.3%]	43.5%; [32.8%, 54.3%]
Week 6	58.4%; [47.7%, 69.1%]	71.0%; [60.7%, 81.3%]
Week 8	65.1%; [54.9%, 75.2%]	84.1%; [75.6%, 92.6%]
Week 12	83.2%; [73.1%, 93.3%]	81.8%; [72.4%, 91.1%]
Patients who reached a reduction from baseline in CPI ^2^, 24 h-API ^1^, and 24 h-WPI ^3^ by ≥50%		
Week 1	4.0%; [0.1%, 8.0%]	6.9%; [1.9%, 11.9%]
Week 2	10.7%; [4.4%, 17.0%]	20.4%; [12.2%, 28.6%]
Week 6	31.1%; [21.5%, 40.7%]	44.9%; [33.9%, 55.9%]
Week 8	41.4%; [31.0%, 51.8%]	60.2%; [48.7%, 71.7%]
Week 12	57.8%; [45.8%, 69.7%]	67.6%; [57.8%, 77.5%]
Patients with CPI ^2^, 24 h-API ^1^, and 24 h-WPI ^3^ not exceeding 10 mm		
Week 6	2.0%; [−0.8%, 4.8%]	11.8%; [5.4%, 18.1%]
Week 8	15.2%; [7.8%, 22.5%]	13.1%; [6.2%, 20.1%]
Week 12	28.9%; [19.6%, 38.2%]	22.0%; [13.7%, 30.2%]
Patients with clinically meaningful improvement (≥30% and/or not exceeding 30 mm) in 4 wk-API ^4^ and 4 wk-WPI ^5^		
Week 8	63.0%; [52.6%, 73.5%]	79.6%; [71.1%, 88.1%]
Week 12	79.6%; [71.0%, 88.2%]	78.6%; [70.3%, 86.9%]
Patients who reached a reduction from baseline in 4 wk-API ^4^ and 4 wk-WPI ^5^ by ≥50%		
Week 8	38.8%; [27.9%, 49.7%]	53.1%; [42.9%, 63.4%]
Week 12	56.4%; [45.5%, 67.3%]	67.3%; [57.3%, 77.2%]
Patients with 4 wk-API ^4^ and 4 wk-WPI ^5^ not exceeding 10 mm		
Week 8	8.5%; [2.5%, 14.4%]	7.8%; [2.3%, 13.4%]
Week 12	17.6%; [9.8%, 25.3%]	14.9%; [7.6%, 22.2%]
DN-4 ^6^		
Week 1	6.9 ± 1.6	6.9 ± 1.6
Week 12	3.6 ± 2.4	3.6 ± 2.5
Week 1 to Week 12 Mean Absolute Change	−3.3; [−3.8, −2.8]	−3.3; [−3.8, −2.8]
Week 1 to Week 12 Relative Difference	−47.5%	−48.0%

^1^ 24 h-API—average pain intensity in the last 24 h. ^2^ CPI—current pain intensity. ^3^ 24 h-WPI—worst pain intensity in the last 24 h. ^4^ 4 wk-API—average pain intensity in the last 4 weeks. ^5^ 4 wk-WPI—worst pain intensity in the last 4 weeks. ^6^ DN-4—Douleur Neuropathique questionnaire. Proportion and 95% confidence interval.

**Table 3:** Mean absolute change in pain from baseline.

	Time Point	Pregabalin Arm (*n* = 99)	Duloxetine Arm (*n* = 102)
CPI ^1^ (mm)	Week 1	−8.7; [−12.1, −5.2]	−9.4; [−13.3, −5.5]
	Week 2	−15.1; [−18.9, −11.3]	−17.8; [−21.5, −14.0]
	Week 6	−22.9; [−27.1, −18.6]	−27.5; [−31.8, −23.3]
	Week 8	−26.7; [−31.2, −22.2]	−32.8; [−37.2, −28.3]
	Week 12	−35.3; [−40.5, −30.0]	−35.0; [−39.2, −30.7]
24 h-API ^2^ (mm)	Week 1	−10.8; [−13.5, −8.1]	−10.8; [−13.7, −7.9]
	Week 2	−16.9; [−20.1, −13.8]	−19.0; [−22.8, −15.2]
	Week 6	−25.5; [−29.1, −22.0]	−29.5; [−33.3, −25.8]
	Week 8	−28.7; [−32.5, −24.8]	−34.8; [−38.5, −31.1]
	Week 12	−37.0; [−41.4, −32.6]	−36.9; [−41.5, −32.3]
24 h-WPI ^3^ (mm)	Week 1	−10.2; [−12.9, −7.5]	−10.7; [−14.1, −7.4]
	Week 2	−16.7; [−19.9, −13.6]	−18.7; [−23.0, −14.3]
	Week 6	−24.5; [−28.5, −20.6]	−31.6; [−36.4, −26.8]
	Week 8	−31.4; [−36.2, −26.6]	−38.2; [−43.5, −32.9]
	Week 12	−41.6; [−46.6, −36.5]	−40.0; [−44.8, −35.2]
4 wk-API ^4^ (mm)	Week 8	−26.2; [−30.0, −22.5]	−32.7; [−36.6, −28.8]
	Week 12	−32.2; [−35.8, −28.7]	−35.7; [−40.4, −31.0]
4 wk-WPI ^5^ (mm)	Week 8	−30.9; [−35.3, −26.5]	−35.4; [−39.9, −30.9]
	Week 12	−38.5; [−42.9, −34.1]	−40.6; [−45.3, −35.8]

^1^ CPI—current pain intensity. ^2^ 24 h-API—average pain intensity in the last 24 h. ^3^ 24 h-WPI—worst pain intensity in the last 24 h. ^4^ 4 wk-API—average pain intensity in the last 4 weeks. ^5^ 4 wk-WPI—worst pain intensity in the last 4 weeks. Mean and 95% confidence interval.

**Table 4:** Adverse events.

Non-Serious Adverse Events (Pregabalin Arm, *n* = 99)	Mild		Moderate		Severe		Total		
Patients with non-serious adverse events	21 (21.2%)		19 (19.2%)		1 (1%)		32 (32.3%)		
SOC/PT	Related	Not related	Related	Not related	Related	Not related	Related	Not related	Total
Nervous system disorders	10 (10.1%)	0 (0%)	10 (10.1%)	0 (0%)	0 (0%)	0 (0%)	18 (18.2%)	0 (0%)	18 (18.2%)
Ear and labyrinth disorders	2 (2%)	0 (0%)	6 (6.1%)	0 (0%)	0 (0%)	0 (0%)	8 (8.1%)	0 (0%)	8 (8.1%)
General disorders and administration site conditions	5 (5.1%)	0 (0%)	2 (2%)	0 (0%)	0 (0%)	0 (0%)	7 (7.1%)	0 (0%)	7 (7.1%)
Gastrointestinal disorders	2 (2%)	0 (0%)	1 (1%)	1 (1%)	0 (0%)	0 (0%)	3 (3%)	1 (1%)	4 (4%)
Psychiatric disorders	2 (2%)	0 (0%)	2 (2%)	0 (0%)	0 (0%)	0 (0%)	4 (4%)	0 (0%)	4 (4%)
Investigations	0 (0%)	2 (2%)	1 (1%)	0 (0%)	0 (0%)	0 (0%)	1 (1%)	2 (2%)	3 (3%)
Metabolism and nutrition disorders	1 (1%)	1 (1%)	1 (1%)	0 (0%)	1 (1%)	0 (0%)	2 (2%)	1 (1%)	3 (3%)
Musculoskeletal and connective tissue disorders	1 (1%)	1 (1%)	1 (1%)	0 (0%)	0 (0%)	0 (0%)	2 (2%)	1 (1%)	3 (3%)
Eye disorders	1 (1%)	0 (0%)	1 (1%)	0 (0%)	0 (0%)	0 (0%)	2 (2%)	0 (0%)	2 (2%)
Skin and subcutaneous tissue disorders	0 (0%)	2 (2%)	0 (0%)	0 (0%)	0 (0%)	0 (0%)	0 (0%)	2 (2%)	2 (2%)
Metabolism and nutrition disorders/gastrointestinal disorders	1 (1%)	0 (0%)	0 (0%)	0 (0%)	0 (0%)	0 (0%)	1 (1%)	0 (0%)	1 (1%)
Vascular disorders	1 (1%)	0 (0%)	0 (0%)	0 (0%)	0 (0%)	0 (0%)	1 (1%)	0 (0%)	1 (1%)
**Non-serious adverse events (duloxetine arm, *n* = 102)**	**Mild**		**Moderate**		**Severe**		**Total**		
Patients with non-serious adverse events	20 (19.6%)		22 (21.6%)		4 (3.9%)		33 (32.4%)		
SOC/PT	Related	Not related	Related	Not related	Related	Not related	Related	Not related	Total
Gastrointestinal disorders	9 (8.8%)	1 (1%)	8 (7.8%)	2 (2%)	3 (2.9%)	0 (0%)	16 (15.7%)	3 (2.9%)	17 (16.7%)
Nervous system disorders	8 (7.8%)	1 (1%)	5 (4.9%)	1 (1%)	1 (1%)	0 (0%)	14 (13.7%)	2 (2%)	16 (15.7%)
General disorders and administration site conditions	0 (0%)	1 (1%)	4 (3.9%)	0 (0%)	0 (0%)	0 (0%)	4 (3.9%)	1 (1%)	5 (4.9%)
Psychiatric disorders	2 (2%)	0 (0%)	3 (2.9%)	0 (0%)	0 (0%)	0 (0%)	5 (4.9%)	0 (0%)	5 (4.9%)
Ear and labyrinth disorders	0 (0%)	0 (0%)	4 (3.9%)	0 (0%)	0 (0%)	0 (0%)	4 (3.9%)	0 (0%)	4 (3.9%)
Metabolism and nutrition disorders	2 (2%)	0 (0%)	1 (1%)	0 (0%)	1 (1%)	0 (0%)	4 (3.9%)	0 (0%)	4 (3.9%)
Skin and subcutaneous tissue disorders	1 (1%)	0 (0%)	2 (2%)	0 (0%)	1 (1%)	0 (0%)	4 (3.9%)	0 (0%)	4 (3.9%)
Cardiac disorders	1 (1%)	0 (0%)	1 (1%)	0 (0%)	0 (0%)	0 (0%)	2 (2%)	0 (0%)	2 (2%)
Eye disorders	1 (1%)	0 (0%)	1 (1%)	0 (0%)	0 (0%)	0 (0%)	2 (2%)	0 (0%)	2 (2%)
Injury, poisoning, and procedural complications	1 (1%)	0 (0%)	1 (1%)	0 (0%)	0 (0%)	0 (0%)	2 (2%)	0 (0%)	2 (2%)
Infections and infestations	0 (0%)	0 (0%)	0 (0%)	1 (1%)	0 (0%)	0 (0%)	0 (0%)	1 (1%)	1 (1%)
Reproductive system and breast disorders	0 (0%)	0 (0%)	1 (1%)	0 (0%)	0 (0%)	0 (0%)	1 (1%)	0 (0%)	1 (1%)

**Table 5:** Summaries of prescribed medication dosage and proportion of patients with compliance exceeding 80%.

		N	Mean ± SD	Median	[Min, Max]	Q1, Q3	Patients with Compliance Exceeding 80%
Pregabalin (mg)	Week 1	99	69.44 ± 32.25	75	[25, 150]	50, 75	/
	Week 2	96	140.89 ± 33.07	150	[50, 300]	150, 150	97.8%; [94.7%, 100.9%]
	Week 6	95	224.21 ± 92.5	150	[150, 600]	150, 300	/
	Week 8	90	256.67 ± 129.65	300	[150, 600]	150, 300	96.8%; [93.1%, 100.5%]
	Week 12	85	294.71 ± 162.76	300	[150, 600]	150, 300	89.3%; [81.7%, 96.9%]
Duloxetine (mg)	Week 1	102	35 ± 11.24	30	[30, 60]	30, 30	/
	Week 2	99	54.55 ± 11.63	60	[30, 60]	60, 60	98.6%; [95.4%, 101.8%]
	Week 6	85	70.59 ± 19.48	60	[60, 120]	60, 90	/
	Week 8	80	76.13 ± 22.36	60	[60, 120]	60, 90	94.5%; [89.9%, 99.2%]
	Week 12	76	80.13 ± 26.1	60	[30, 120]	60, 97.5	92.7%; [85.7%, 99.7%]

## Data Availability

Data are contained within the article and the Supplementary Materials.

## Supplementary Materials

The following supporting information can be downloaded at https://www.mdpi.com/article/10.3390/ph16071017/s1, Table S1: Inclusion and exclusion criteria for clinical trial, Figure S1: Treatment protocol with the plan of visits with possible daily doses of investigational medicinal product (IMP), Table S2: Treatment-emergent non-serious adverse events in pregabalin arm: number and proportion of patients with events, Table S3: Treatment-emergent non-serious adverse events in duloxetine arm: number and proportion of patients with events. Reference [36] is cited in Supplementary Materials.
