# Supplementary material for: Efficacy of Pregabalin and Duloxetine in Patients with Painful Diabetic Peripheral Neuropathy (PDPN): A Multi-Centre Phase IV Clinical Trial—BLOSSOM
Source: Pharmaceuticals (Basel). 2023 Jul 18;16(7):1017. doi: 10.3390/ph16071017 (PMC10386018; doi:10.3390/ph16071017)
Supplement: Supplementary file 1 [file pharmaceuticals-16-01017-s001.zip › pharmaceuticals-2396689-supplementary.pdf]

Supplementary Table S1. Inclusion and exclusion criteria for clinical trial

**Inclusion criteria**	**Exclusion criteria**
Female and male patients 18–85 years of age.	Patients who took PDPN medication and/or analgesics on a day of baseline visit.
Patients with a history of type 2 diabetes mellitus according to the American Diabetes Association (ADA).	Patients with a known hypersensitivity to duloxetine, pregabalin, paracetamol or tramadol or any of the inactive ingredients or who have any contraindication for the use of duloxetine, pregabalin, paracetamol or tramadol.
Patients with a diagnosis of painful diabetic peripheral neuropathy (PDPN) caused by type 2 diabetes mellitus based on DN4 ≥4.	Patients with a history of inadequate pain response (pain reduced was equal or less than 30%) to:<br>1. pregabalin at maximum allowed treatment daily dose of 600 mg,<br>2. duloxetine at maximum allowed treatment daily dose of 120 mg,<br>3. venlafaxine at maximum allowed treatment daily dose of 375 mg,<br>4. gabapentin at daily treatment dose of more than 1800 mg,<br>5. amitriptilin at maximum allowed treatment daily dose of 150 mg.
Patients whose average pain intensity in PDPN in the last 24 hours (measured by VAS), evaluated at baseline visit, is equal or more than 40 mm (0 mm ='no pain' and 100 mm ='worst possible pain').	Patients who are currently being treated with a daily dose that exceeds:<br>1. 150 mg of pregabalin<br>2. 60 mg of duloxetine,<br>3. 150 mg of venlafaxine,<br>4. 600 mg of gabapentin.
Ability to adhere to trial protocol.	Patients with an uncontrolled type 2 diabetes mellitus.
Written informed consent.	The average scores of less than 20 on MoCA.
	Have any other type of neuropathic pain, contrasted to PDPN.
	Evidence of another cause of distal polyneuropathy other than diabetic.
	Have a serious (evaluated by physician) unstable cardiovascular (e.g. uncontrolled hypertension), hepatic, renal, respiratory, ophthalmologic, gastrointestinal, or hematologic illness, symptomatic peripheral vascular disease, malignant disease or other medical condition that could lead to hospitalisation during the course of the trial.
	Known or suspected alcohol or drug abuse or addiction (excluding nicotine and caffeine).
	Patients with a history of depression (less than one year after completing the last medical treatment), mania, bipolar disorder, psychosis or schizophrenia.
	Pregnancy, lactation and women of childbearing potential without highly effective* or at least acceptable** contraception (according to the *Recommendations related to contraception and pregnancy testing in clinical trials*).[36]
	Patients with a history of epilepsy, stroke or neurodegenerative disease.

	Patients taking monoamine oxidase (MAO) inhibitors or those within one year of withdrawal.
	Acute liver injury (such as hepatitis) or severe cirrhosis (Child-Pugh Class C).
	Patients with suspected restless leg syndrome (RLS).
	Abnormal thyroid-stimulating hormone (TSH) concentrations (according to the references value of the local laboratory).
	Vitamin B12 and folic acid deficiency (according to the reference values of the local laboratory).
	Surgical procedures planned to occur during trial (patients may be re-screened following completion of and recovery from the surgical procedure).
	Concomitant treatment that might influence the final therapeutic effect of the tested active substances including non-medical treatments.
	Patients who under the opinion of the investigator will not be compliant to the treatment or will not be able to finish the trial for any other reason.

*Highly effective contraception is:

- combined (estrogen and progestogen containing) hormonal contraception associated with inhibition of ovulation (oral, intravaginal, transdermal),
- progestogen-only hormonal contraception associated with inhibition of ovulation (oral, injectable, implantable),
- intrauterine device (IUD),
- intrauterine hormone-releasing system (IUS),
- bilateral tubal occlusion,
- vasectomised partner,
- sexual abstinence.

**Acceptable contraception is:

- progestogen-only oral hormonal contraception, where inhibition of ovulation is not the primary mode of action,
- male or female condom with or without spermicide,
- cap, diaphragm or sponge with spermicide.

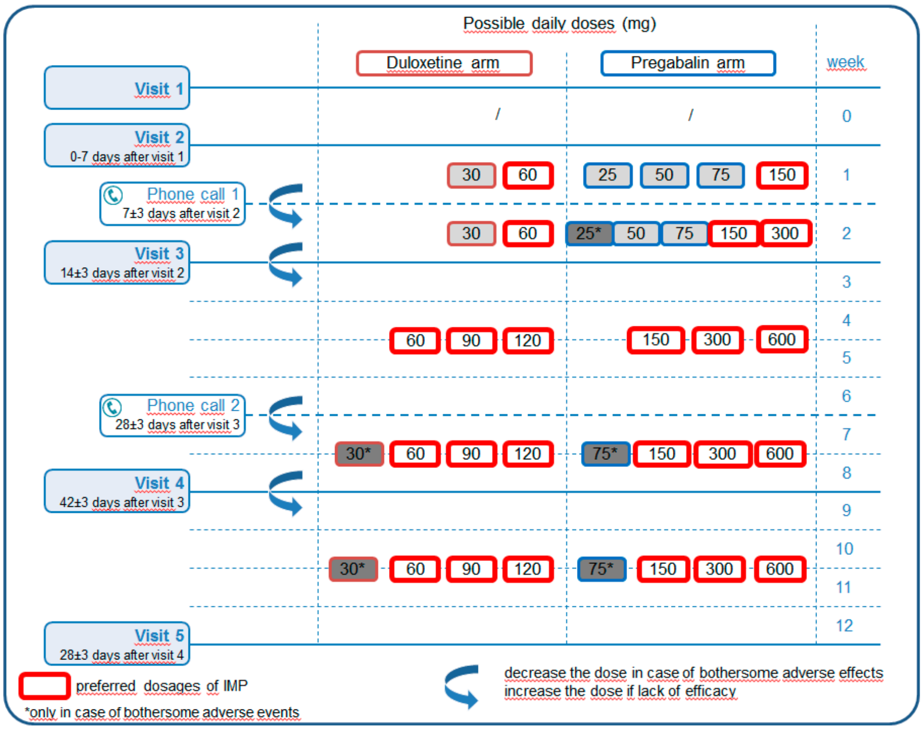

Supplementary Figure S1. Treatment protocol with the plan of visits with possible daily doses of investigational medicinal product (IMP).

Supplementary Table S2. Treatment emergent non-serious adverse events in pregabalin arm: number and proportion of patients with events.

**Non-serious adverse events (pregabalin arm, n = 99)**	**Mild**		**Moderate**		**Severe**		**Total**		
Patients with non-serious adverse events	21 (21.2%)		19 (19.2%)		1 (1%)		32 (32.3%)		
SOC/PT	Related	Not related	Related	Not related	Related	Not related	Related	Not related	Total
**Nervous system disorders**	**10 (10.1%)**	**0 (0%)**	**10 (10.1%)**	**0 (0%)**	**0 (0%)**	**0 (0%)**	**18 (18.2%)**	**0 (0%)**	**18 (18.2%)**
Somnolence	5 (5.1%)	0 (0%)	5 (5.1%)	0 (0%)	0 (0%)	0 (0%)	9 (9.1%)	0 (0%)	9 (9.1%)
Dizziness	3 (3%)	0 (0%)	4 (4%)	0 (0%)	0 (0%)	0 (0%)	7 (7.1%)	0 (0%)	7 (7.1%)
Balance disorder	1 (1%)	0 (0%)	1 (1%)	0 (0%)	0 (0%)	0 (0%)	2 (2%)	0 (0%)	2 (2%)
Headache	1 (1%)	0 (0%)	1 (1%)	0 (0%)	0 (0%)	0 (0%)	2 (2%)	0 (0%)	2 (2%)
Burning sensation	1 (1%)	0 (0%)	0 (0%)	0 (0%)	0 (0%)	0 (0%)	1 (1%)	0 (0%)	1 (1%)
Disturbance in attention	1 (1%)	0 (0%)	0 (0%)	0 (0%)	0 (0%)	0 (0%)	1 (1%)	0 (0%)	1 (1%)
**Ear and labyrinth disorders**	**2 (2%)**	**0 (0%)**	**6 (6.1%)**	**0 (0%)**	**0 (0%)**	**0 (0%)**	**8 (8.1%)**	**0 (0%)**	**8 (8.1%)**

**Non-serious adverse events (pregabalin arm, n = 99)**	**Mild**		**Moderate**		**Severe**		**Total**		
Patients with non-serious adverse events	21 (21.2%)		19 (19.2%)		1 (1%)		32 (32.3%)		
SOC/PT	Related	Not related	Related	Not related	Related	Not related	Related	Not related	Total
Vertigo	2 (2%)	0 (0%)	5 (5.1%)	0 (0%)	0 (0%)	0 (0%)	7 (7.1%)	0 (0%)	7 (7.1%)
Tinnitus	0 (0%)	0 (0%)	1 (1%)	0 (0%)	0 (0%)	0 (0%)	1 (1%)	0 (0%)	1 (1%)
**General disorders and administration site conditions**	**5 (5.1%)**	**0 (0%)**	**2 (2%)**	**0 (0%)**	**0 (0%)**	**0 (0%)**	**7 (7.1%)**	**0 (0%)**	**7 (7.1%)**
Peripheral oedema	2 (2%)	0 (0%)	0 (0%)	0 (0%)	0 (0%)	0 (0%)	2 (2%)	0 (0%)	2 (2%)
Peripheral swelling	1 (1%)	0 (0%)	1 (1%)	0 (0%)	0 (0%)	0 (0%)	2 (2%)	0 (0%)	2 (2%)
Asthenia	1 (1%)	0 (0%)	0 (0%)	0 (0%)	0 (0%)	0 (0%)	1 (1%)	0 (0%)	1 (1%)
Fatigue	0 (0%)	0 (0%)	1 (1%)	0 (0%)	0 (0%)	0 (0%)	1 (1%)	0 (0%)	1 (1%)
Oedema	1 (1%)	0 (0%)	0 (0%)	0 (0%)	0 (0%)	0 (0%)	1 (1%)	0 (0%)	1 (1%)
**Gastrointestinal disorders**	**2 (2%)**	**0 (0%)**	**1 (1%)**	**1 (1%)**	**0 (0%)**	**0 (0%)**	**3 (3%)**	**1 (1%)**	**4 (4%)**
Nausea	2 (2%)	0 (0%)	0 (0%)	0 (0%)	0 (0%)	0 (0%)	2 (2%)	0 (0%)	2 (2%)
Abdominal distension	0 (0%)	0 (0%)	1 (1%)	0 (0%)	0 (0%)	0 (0%)	1 (1%)	0 (0%)	1 (1%)
Diarrhoea	0 (0%)	0 (0%)	0 (0%)	1 (1%)	0 (0%)	0 (0%)	0 (0%)	1 (1%)	1 (1%)
**Psychiatric disorders**	**2 (2%)**	**0 (0%)**	**2 (2%)**	**0 (0%)**	**0 (0%)**	**0 (0%)**	**4 (4%)**	**0 (0%)**	**4 (4%)**
Confusional state	1 (1%)	0 (0%)	1 (1%)	0 (0%)	0 (0%)	0 (0%)	2 (2%)	0 (0%)	2 (2%)
Insomnia	0 (0%)	0 (0%)	1 (1%)	0 (0%)	0 (0%)	0 (0%)	1 (1%)	0 (0%)	1 (1%)
Mood altered	1 (1%)	0 (0%)	0 (0%)	0 (0%)	0 (0%)	0 (0%)	1 (1%)	0 (0%)	1 (1%)
**Investigations**	**0 (0%)**	**2 (2%)**	**1 (1%)**	**0 (0%)**	**0 (0%)**	**0 (0%)**	**1 (1%)**	**2 (2%)**	**3 (3%)**
Blood pressure increased	0 (0%)	1 (1%)	0 (0%)	0 (0%)	0 (0%)	0 (0%)	0 (0%)	1 (1%)	1 (1%)
Platelet count decreased	0 (0%)	1 (1%)	0 (0%)	0 (0%)	0 (0%)	0 (0%)	0 (0%)	1 (1%)	1 (1%)
Weight increased	0 (0%)	0 (0%)	1 (1%)	0 (0%)	0 (0%)	0 (0%)	1 (1%)	0 (0%)	1 (1%)
**Metabolism and nutrition disorders**	**1 (1%)**	**1 (1%)**	**1 (1%)**	**0 (0%)**	**1 (1%)**	**0 (0%)**	**2 (2%)**	**1 (1%)**	**3 (3%)**
Hyperglycaemia	1 (1%)	1 (1%)	0 (0%)	0 (0%)	0 (0%)	0 (0%)	1 (1%)	1 (1%)	2 (2%)
Increased appetite	0 (0%)	0 (0%)	1 (1%)	0 (0%)	1 (1%)	0 (0%)	1 (1%)	0 (0%)	1 (1%)

**Non-serious adverse events (pregabalin arm, n = 99)**	**Mild**		**Moderate**		**Severe**		**Total**		
Patients with non-serious adverse events	21 (21.2%)		19 (19.2%)		1 (1%)		32 (32.3%)		
SOC/PT	Related	Not related	Related	Not related	Related	Not related	Related	Not related	Total
**Musculoskeletal and connective tissue disorders**	**1 (1%)**	**1 (1%)**	**1 (1%)**	**0 (0%)**	**0 (0%)**	**0 (0%)**	**2 (2%)**	**1 (1%)**	**3 (3%)**
Joint swelling	0 (0%)	1 (1%)	0 (0%)	0 (0%)	0 (0%)	0 (0%)	0 (0%)	1 (1%)	1 (1%)
Muscle spasms	0 (0%)	0 (0%)	1 (1%)	0 (0%)	0 (0%)	0 (0%)	1 (1%)	0 (0%)	1 (1%)
Spinal pain	1 (1%)	0 (0%)	0 (0%)	0 (0%)	0 (0%)	0 (0%)	1 (1%)	0 (0%)	1 (1%)
**Eye disorders**	**1 (1%)**	**0 (0%)**	**1 (1%)**	**0 (0%)**	**0 (0%)**	**0 (0%)**	**2 (2%)**	**0 (0%)**	**2 (2%)**
Vision blurred	1 (1%)	0 (0%)	1 (1%)	0 (0%)	0 (0%)	0 (0%)	2 (2%)	0 (0%)	2 (2%)
**Skin and subcutaneous tissue disorders**	**0 (0%)**	**2 (2%)**	**0 (0%)**	**0 (0%)**	**0 (0%)**	**0 (0%)**	**0 (0%)**	**2 (2%)**	**2 (2%)**
Dandruff	0 (0%)	1 (1%)	0 (0%)	0 (0%)	0 (0%)	0 (0%)	0 (0%)	1 (1%)	1 (1%)
Rash	0 (0%)	1 (1%)	0 (0%)	0 (0%)	0 (0%)	0 (0%)	0 (0%)	1 (1%)	1 (1%)
**Metabolism and nutrition disorders / gastrointestinal disorders**	**1 (1%)**	**0 (0%)**	**0 (0%)**	**0 (0%)**	**0 (0%)**	**0 (0%)**	**1 (1%)**	**0 (0%)**	**1 (1%)**
Increased appetite / constipation	1 (1%)	0 (0%)	0 (0%)	0 (0%)	0 (0%)	0 (0%)	1 (1%)	0 (0%)	1 (1%)
**Vascular disorders**	**1 (1%)**	**0 (0%)**	**0 (0%)**	**0 (0%)**	**0 (0%)**	**0 (0%)**	**1 (1%)**	**0 (0%)**	**1 (1%)**
Hypertension	1 (1%)	0 (0%)	0 (0%)	0 (0%)	0 (0%)	0 (0%)	1 (1%)	0 (0%)	1 (1%)

Supplementary Table S3. Treatment emergent non-serious adverse events in duloxetine arm: number and proportion of patients with events.

**Non-serious adverse events (duloxetine arm, n = 102)**	**Mild**		**Moderate**		**Severe**		**Total**		
Patients with non-serious adverse events	20 (19.6%)		22 (21.6%)		4 (3.9%)		33 (32.4%)		
SOC/PT	Related	Not related	Related	Not related	Related	Not related	Related	Not related	Total
**Gastrointestinal disorders**	**9 (8.8%)**	**1 (1%)**	**8 (7.8%)**	**2 (2%)**	**3 (2.9%)**	**0 (0%)**	**16 (15.7%)**	**3 (2.9%)**	**17 (16.7%)**
Nausea	7 (6.9%)	0 (0%)	3 (2.9%)	0 (0%)	2 (2%)	0 (0%)	10 (9.8%)	0 (0%)	10 (9.8%)
Vomiting	0 (0%)	1 (1%)	3 (2.9%)	0 (0%)	1 (1%)	0 (0%)	4 (3.9%)	1 (1%)	5 (4.9%)
Dry mouth	1 (1%)	0 (0%)	3 (2.9%)	0 (0%)	0 (0%)	0 (0%)	4 (3.9%)	0 (0%)	4 (3.9%)
Abdominal pain	0 (0%)	1 (1%)	0 (0%)	1 (1%)	1 (1%)	0 (0%)	1 (1%)	2 (2%)	3 (2.9%)
Diarrhoea	1 (1%)	0 (0%)	0 (0%)	1 (1%)	0 (0%)	0 (0%)	1 (1%)	1 (1%)	2 (2%)
Constipation	1 (1%)	0 (0%)	0 (0%)	0 (0%)	0 (0%)	0 (0%)	1 (1%)	0 (0%)	1 (1%)
Salivary hypersecretion	0 (0%)	0 (0%)	0 (0%)	1 (1%)	0 (0%)	0 (0%)	0 (0%)	1 (1%)	1 (1%)
**Nervous system disorders**	**8 (7.8%)**	**1 (1%)**	**5 (4.9%)**	**1 (1%)**	**1 (1%)**	**0 (0%)**	**14 (13.7%)**	**2 (2%)**	**16 (15.7%)**
Dizziness	4 (3.9%)	1 (1%)	3 (2.9%)	0 (0%)	0 (0%)	0 (0%)	7 (6.9%)	1 (1%)	8 (7.8%)
Somnolence	4 (3.9%)	0 (0%)	2 (2%)	0 (0%)	1 (1%)	0 (0%)	7 (6.9%)	0 (0%)	7 (6.9%)
Headache	0 (0%)	0 (0%)	2 (2%)	0 (0%)	1 (1%)	0 (0%)	3 (2.9%)	0 (0%)	3 (2.9%)
Balance disorder	1 (1%)	0 (0%)	0 (0%)	0 (0%)	0 (0%)	0 (0%)	1 (1%)	0 (0%)	1 (1%)
Dysgeusia	1 (1%)	0 (0%)	0 (0%)	0 (0%)	0 (0%)	0 (0%)	1 (1%)	0 (0%)	1 (1%)
Tremor	0 (0%)	0 (0%)	0 (0%)	1 (1%)	0 (0%)	0 (0%)	0 (0%)	1 (1%)	1 (1%)
**General disorders and administration site conditions**	**0 (0%)**	**1 (1%)**	**4 (3.9%)**	**0 (0%)**	**0 (0%)**	**0 (0%)**	**4 (3.9%)**	**1 (1%)**	**5 (4.9%)**
Chest pain	0 (0%)	0 (0%)	2 (2%)	0 (0%)	0 (0%)	0 (0%)	2 (2%)	0 (0%)	2 (2%)
Fatigue	0 (0%)	0 (0%)	1 (1%)	0 (0%)	0 (0%)	0 (0%)	1 (1%)	0 (0%)	1 (1%)
Pain	0 (0%)	0 (0%)	1 (1%)	0 (0%)	0 (0%)	0 (0%)	1 (1%)	0 (0%)	1 (1%)
Pyrexia	0 (0%)	1 (1%)	0 (0%)	0 (0%)	0 (0%)	0 (0%)	0 (0%)	1 (1%)	1 (1%)
**Psychiatric disorders**	**2 (2%)**	**0 (0%)**	**3 (2.9%)**	**0 (0%)**	**0 (0%)**	**0 (0%)**	**5 (4.9%)**	**0 (0%)**	**5 (4.9%)**
Insomnia	0 (0%)	0 (0%)	1 (1%)	0 (0%)	0 (0%)	0 (0%)	1 (1%)	0 (0%)	1 (1%)
Loss of libido	1 (1%)	0 (0%)	0 (0%)	0 (0%)	0 (0%)	0 (0%)	1 (1%)	0 (0%)	1 (1%)
Nightmare	0 (0%)	0 (0%)	1 (1%)	0 (0%)	0 (0%)	0 (0%)	1 (1%)	0 (0%)	1 (1%)
Restlessness	1 (1%)	0 (0%)	0 (0%)	0 (0%)	0 (0%)	0 (0%)	1 (1%)	0 (0%)	1 (1%)
Sleep disorder	0 (0%)	0 (0%)	1 (1%)	0 (0%)	0 (0%)	0 (0%)	1 (1%)	0 (0%)	1 (1%)
**Ear and labyrinth disorders**	**0 (0%)**	**0 (0%)**	**4 (3.9%)**	**0 (0%)**	**0 (0%)**	**0 (0%)**	**4 (3.9%)**	**0 (0%)**	**4 (3.9%)**

**Non-serious adverse events (duloxetine arm, n = 102)**	**Mild**		**Moderate**		**Severe**		**Total**		
Patients with non-serious adverse events	20 (19.6%)		22 (21.6%)		4 (3.9%)		33 (32.4%)		
SOC/PT	Related	Not related	Related	Not related	Related	Not related	Related	Not related	Total
Tinnitus	0 (0%)	0 (0%)	2 (2%)	0 (0%)	0 (0%)	0 (0%)	2 (2%)	0 (0%)	2 (2%)
Vertigo	0 (0%)	0 (0%)	2 (2%)	0 (0%)	0 (0%)	0 (0%)	2 (2%)	0 (0%)	2 (2%)
Inner ear disorder	0 (0%)	0 (0%)	1 (1%)	0 (0%)	0 (0%)	0 (0%)	1 (1%)	0 (0%)	1 (1%)
**Metabolism and nutrition disorders**	**2 (2%)**	**0 (0%)**	**1 (1%)**	**0 (0%)**	**1 (1%)**	**0 (0%)**	**4 (3.9%)**	**0 (0%)**	**4 (3.9%)**
Decreased appetite	2 (2%)	0 (0%)	1 (1%)	0 (0%)	1 (1%)	0 (0%)	4 (3.9%)	0 (0%)	4 (3.9%)
Hyperglycaemia	0 (0%)	0 (0%)	1 (1%)	0 (0%)	0 (0%)	0 (0%)	1 (1%)	0 (0%)	1 (1%)
**Skin and subcutaneous tissue disorders**	**1 (1%)**	**0 (0%)**	**2 (2%)**	**0 (0%)**	**1 (1%)**	**0 (0%)**	**4 (3.9%)**	**0 (0%)**	**4 (3.9%)**
Hyperhidrosis	1 (1%)	0 (0%)	2 (2%)	0 (0%)	1 (1%)	0 (0%)	4 (3.9%)	0 (0%)	4 (3.9%)
**Cardiac disorders**	**1 (1%)**	**0 (0%)**	**1 (1%)**	**0 (0%)**	**0 (0%)**	**0 (0%)**	**2 (2%)**	**0 (0%)**	**2 (2%)**
Palpitations	1 (1%)	0 (0%)	1 (1%)	0 (0%)	0 (0%)	0 (0%)	2 (2%)	0 (0%)	2 (2%)
**Eye disorders**	**1 (1%)**	**0 (0%)**	**1 (1%)**	**0 (0%)**	**0 (0%)**	**0 (0%)**	**2 (2%)**	**0 (0%)**	**2 (2%)**
Diplopia	0 (0%)	0 (0%)	1 (1%)	0 (0%)	0 (0%)	0 (0%)	1 (1%)	0 (0%)	1 (1%)
Vision blurred	1 (1%)	0 (0%)	0 (0%)	0 (0%)	0 (0%)	0 (0%)	1 (1%)	0 (0%)	1 (1%)
Visual impairment	0 (0%)	0 (0%)	1 (1%)	0 (0%)	0 (0%)	0 (0%)	1 (1%)	0 (0%)	1 (1%)
**Injury, poisoning and procedural complications**	**1 (1%)**	**0 (0%)**	**1 (1%)**	**0 (0%)**	**0 (0%)**	**0 (0%)**	**2 (2%)**	**0 (0%)**	**2 (2%)**
Fall	1 (1%)	0 (0%)	1 (1%)	0 (0%)	0 (0%)	0 (0%)	2 (2%)	0 (0%)	2 (2%)
**Infections and infestations**	**0 (0%)**	**0 (0%)**	**0 (0%)**	**1 (1%)**	**0 (0%)**	**0 (0%)**	**0 (0%)**	**1 (1%)**	**1 (1%)**
Bronchitis	0 (0%)	0 (0%)	0 (0%)	1 (1%)	0 (0%)	0 (0%)	0 (0%)	1 (1%)	1 (1%)
**Reproductive system and breast disorders**	**0 (0%)**	**0 (0%)**	**1 (1%)**	**0 (0%)**	**0 (0%)**	**0 (0%)**	**1 (1%)**	**0 (0%)**	**1 (1%)**
Erectile dysfunction	0 (0%)	0 (0%)	1 (1%)	0 (0%)	0 (0%)	0 (0%)	1 (1%)	0 (0%)	1 (1%)
